# Characterization and functional analysis of *hsp18.3* gene in the red flour beetle, *Tribolium castaneum*

**DOI:** 10.1111/1744-7917.12543

**Published:** 2017-12-07

**Authors:** Jia Xie, Xing‐Xing Hu, Meng‐Fan Zhai, Xiao‐Juan Yu, Xiao‐Wen Song, Shan‐Shan Gao, Wei Wu, Bin Li

**Affiliations:** ^1^ Jiangsu Key Laboratory for Biodiversity and Biotechnology, College of Life Sciences Nanjing Normal University Nanjing China

**Keywords:** development, reproduction, stress, *Tchsp18.3*, *Tribolium castaneum*

## Abstract

Small heat shock proteins (sHSPs) are diverse and mainly function as molecular chaperones to protect organisms and cells from various stresses. In this study, *hsp18.3*, one *Tribolium castaneum* species‐specific *shsp*, has been identified. Quantitative real‐time polymerase chain reaction illustrated that *Tchsp18.3* is expressed in all developmental stages, and is highly expressed at early pupal and late adult stages, while it is highly expressed in ovary and fat body at the adult period. Moreover, it was up‐regulated 4532 ± 396‐fold in response to enhanced heat stress but not to cold stress; meanwhile the lifespan of adults in ds‐*Tchsp18.3* group reduced by 15.8% from control group under starvation. Laval RNA interference (RNAi) of *Tchsp18.3* caused 86.1% ± 4.5% arrested pupal eclosion and revealed that *Tchsp18.3* played an important role in insect development. In addition, parental RNAi of *Tchsp18.3* reduced the oviposition amount by 94.7%. These results suggest that *Tchsp18.3* is not only essential for the resistance to heat and starvation stress, but also is critical for normal development and reproduction in *T. castaneum*.

## Introduction

Heat shock proteins (HSPs) were first discovered in 1962 by Ritossa and his co‐workers in overheated *Drosophila melanogaster* larvae (Ritossa, 1962), and subsequent studies found they are a set of evolutionarily conserved proteins present in virtually all living organisms, from bacteria to humans. It has been shown that most HSPs have strong cytoprotective effects and behave as molecular chaperones for other cellular proteins (Garrido *et al*., 2006). HSPs are commonly classified into five families based on their molecular weight and homologous relationship: HSP100, HSP90, HSP70, HSP60 and the small heat shock proteins (sHSPs) (Kim *et al*., 1998). Mainly because of the wide molecular mass range from 12 to 43 kDa and low homology, compared to other families of HSPs, sHSPs exhibit a greater variation in sequence, structure, size and function (Franck *et al*., 2004).

However, there are still some common characteristics in the structure and function of sHSPs, which have a relatively conserved α‐crystalling domain spanning about 80∼100 amino acid residues, located near the C‐terminal region (Caspers *et al*., 1995; Fu *et al*., 2006; Perez‐Morales & Espinoza, 2015). In the secondary structure, sHSP monomers are enriched in *β*‐strands organized in a *β*‐sheet sandwich responsible for dimer formation (Sun & MacRae, 2005). Most sHSPs display chaperone‐like activities, helping the unfolding proteins maintain their correct states, binding to denatured proteins and preventing irreversible protein aggregation during stresses, such as extreme temperatures, oxidation and heavy metals exposure (Haslbeck *et al*., 2005: Chen & Zhang, 2015). sHSPs have also been involved in a variety of cellular activities, including organization of the cytoskeletal integrity, maintaining signal transduction, modulating membrane lipid polymorphism (Arrigo, 2000; Tsvetkova *et al*., 2002; Bakthisaran *et al*., 2015; Haslbeck & Vierling, 2015). These proteins additionally participate in many other physiological processes, such as the regulation of cell cycle and differentiation, interfering with apoptosis and defending against diseases (Arrigo, 2000).

Recent studies showed that the sHSPs in insects also play various roles. For instance, in *Apis cerana cerana*, *hsp22.6* was significantly up‐regulated by abiotic stresses, such as temperature (4°C, 16°C, 42°C), pesticides (cyhalothrin, pyridaben), oxidative stress (UV, H_2_O_2_) and heavy metals (CdCl_2_), as well as biotic stress, such as 20‐hydroxyecdysone (20E), *Ascosphaera apis* treatment (Zhang *et al*., 2014b). In *D. melanogaster*, over‐expressing the mitochondrial *hsp22* increased the resistance to oxidative stress and extended lifespan by 32%. The longevity phenotype of a strain obtained by P‐element jump‐out was examined. It was found that the flies which did not express mitochondrial *hsp22* led a 40% decrease in lifespan, and these flies died faster and displayed a decrease of 30% in locomotor activity compared with controls (Morrow *et al*., 2004). In addition, *hsp21.3* which increased the expression from neo‐larval to pupal stage may play a critical role in the development of *Grapholita molesta*, and it was the most markedly up‐regulated one together with five other *hsps* during early diapause (Zhang *et al*., 2015).

In the red flour beetle, *Tribolium castaneum* (Coleoptera: Tenebrionidae), which has long been used as an important model organism for insect development, evolution, comparative genomics and pest science (Richards *et al*., 2008; Schroeder *et al*., 2008), ten *shsps* have been identified from the genome, and eight of them were *T. castaneum* species‐specific *shsps* (Li *et al*., 2009). *Hsp18.3*, one *T. castaneum* species‐specific *shsp*, was also mentioned as *hsp27* (XM_969274) in *Tribolium* (Altincicek *et al*., 2008); here we called it *Tchsp18.3* based on its predicted molecular weight (Li *et al*., 2009). Quantitative real‐time polymerase chain reaction (real‐time qPCR) analysis of transcriptional levels of this species‐specific *shsp* were up‐regulated when the beetles were in response to septic wounding, heat shock and UV‐A exposure, suggested that it was involved in immunity and stress responses (Altincicek *et al*., 2008; Sang *et al*., 2012). Additionally, when cell line BCIRL‐TcA‐CLG1 of *T. castaneum* was treated with heat shock, increased salinity, acidic pH and UV‐A light, *hsp18.3* (*hsp27*) seems to be the most affected by 40°C and UV light other than *hsp68a* and *hsp83* (Garcia‐Reina *et al*., 2017). *Hsp18*.3 seems to be an important one in terms of cell growth and viability of *T. castaneum*, but there are no studies carried out with its systematic functions in *T. castaneum*, and whether it has a functional differentiation is still unknown. Here, we further explore the diverse functions of *hsp18.3* in *T. castaneum*, and provide experimental evidence and better understanding of the functions of insect *shsps*.

## Materials and methods

### Experimental insects

The Georgia‐1 (GA‐1) strain of *T. castaneum* was reared at 30 °C and 40% relative humidity in 5% yeasted flour under standard conditions (Haliscak & Beeman, 1983; Li *et al*., 2011).

### Bioinformatic analysis of gene structure

The sHSPs sequences were obtained from Beetlebase (http://www.beetlebase.org/), FlyBase (http://flybase.org/), BeeBase (http://hymenopteragenome.org/beebase/), Silkworm Genome Database (http://silkworm.genomics.org.cn/), VectorBase (http://www.vectorbase.org/index.php). Sequences were aligned with ClustalW2 (http://www.ebi.ac.uk/Tools/msa/clustalw2/) and input to Boxshade program (http://www.ch.embnet.org/software/BOX_form.html). Genes structural prediction were analyzed online using PredictProtein (http://www.predictprotein.org/) and InterProScan (http://www.ebi.ac.uk/Tools/InterProScan/).

### Quantitative real‐time PCR analysis

Total RNAs from pools of three individuals were extracted at each of the following developmental stages: early eggs (EE, 1 day old), late eggs (LE, 3 days old), early larvae (EL, 1 day old), late larvae (LL, last‐instar larvae), early pupae (EP, 1 day old), late pupae (LP, 5 days old), early adults (EA, 1 day old) and late adults (LA, 1 week old) with the RNAiso^TM^Plus reagent (TaKaRa, Kyoto, Japan). In addition, total RNAs from pools were extracted from various tissues of late adults: head, epidermis, gut, fat body, accessory gland, testis and ovary. Reverse transcription was performed using 1 μg total RNA. Real‐time qPCR was performed to check the temporal and spatial expression patterns with FastStart Universal SYBR Green Master (Roche, Indianapolis, IN, USA) following the manufacturer's instructions. The data were expressed here as the relative messenger RNA (mRNA) levels normalized to ribosomal protein S3 (*rps3*) in the same complementary DNA (cDNA) samples, using the 2^−△△^ ${}^{C_{T}}$ method (Livak & Schmittgen, 2001). The primers are listed in Table 1.

**Table 1:** Primers used in this study

Primer name	Primer sequence (5′→3′)
Real time quantitative PCR	
*Tchsp18.3*	CAGGACCATGGGTCTAGTGATA
	TCCCATCGCCAACCTTCG
Double‐stranded RNA	
*Tchsp18.3*	TAATACGACTCACTATAGGGCGACCAGCTCATCGTTTCCT
	TAATACGACTCACTATAGGGTTGTTGCACCGCTGGTGTA

### RNA interference (RNAi)

Double‐stranded RNA (dsRNA) primers containing gene‐specific sequences and the T7 polymerase promoter (TAATACGACTCACTATAGGG) at the 5′‐end of both the sense primer and anti‐sense primer were used (Table 1). The PCR products were used as templates for dsRNA synthesis with the TranscriptAid^TM^ T7 High Yield Transcription Kit (Fermentas, Vilnius, Lithuania). In addition, dsRNA was treated with DNase I and purified using a chloroform extraction followed by ethanol precipitation and dissolved in nuclease‐free water to a concentration of 2 μg/μL. The quality of dsRNA was checked by running on an agarose gel electrophoresis and the concentration was measured using NanoDrop 2000 spectrophotometer (Thermo Fisher Scientific Inc., Waltham, MA, USA).

To explore the effects of *Tchsp18.3* on the development of *T. castaneum*, about 200 ng of dsRNA in 200 nL solution was injected into each late larva (∼20 days old) (Tomoyasu & Denell, 2004). Negative controls consisted of non‐injection (wild type group, WT) or injection of an equal volume of buffer only (IB group). Since knocking down *Tchsp18.3* in the late larval stage induced pupal mortality significantly, the parental RNAi experiment consisted of pupal injection, which did not cause any detectable defects in adult eclosion, was carried out to explore the effects of *Tchsp18.3* on reproduction and starvation tolerance (Bucher *et al*., 2002; Sang *et al*., 2016). Mortality occurring less than 5 days after injection was attributed to injection injury rather than to target transcript knockdown (Begum *et al*., 2009). On the 6th day after dsRNA injection, insects were used to detect whether RNAi is effective. Every group had about 30 individuals. Three biological replications were carried out for each experiment.

### Temperature and starvation stress treatment

To examine whether *Tchsp18.3* is in response to thermal and cold stress, late larvae were reared at 45°C and 4°C, with an ambient temperature of 25°C serving as control, then the samples were collected after the insects were treated with 45°C, 25°C or 4°C for 1 h, 2 h, 4 h and 12 h, respectively. Thereafter, the expression level of *Tchsp18.3* was detected.

For the starvation tolerance assay, parental RNAi was carried out. Then the adults (1 day old) in the WT group, IB group and ds‐*Tchsp18.3* group were kept without food and the expression levels of *Tchsp18.3* and the survival rates were investigated every day. Three biological replications were carried out for the experiments.

### Behavior analysis

Larval injections were followed by the observation of the noticeable morphological defects and mortality. Parental RNAi experiments were followed by the assessment of oviposition rates. Individuals were utilized for single pair mattings (10 to 15 pairs, respectively). Three‐day ovipositions (13‐day‐old to 15‐day‐old females) were collected, counted and held for hatch‐rate measurement and their development observed. Numbers of offspring were counted 15 days after the eggs were collected.

### Statistical analysis

The data analysis was done using SPSS version 13.0 by one‐way analysis of variance, followed by Tukey's Honestly Significant Difference test. All the data are presented as the mean ± SE. Error bars represent standard errors among three biological replications. *P*‐value <0.05 was regarded as statistically significant, while *P*‐value <0.001 was considered extreme significance.

## Results

### Identification of hsp18.3 from T. castaneum

**Figure 1:**
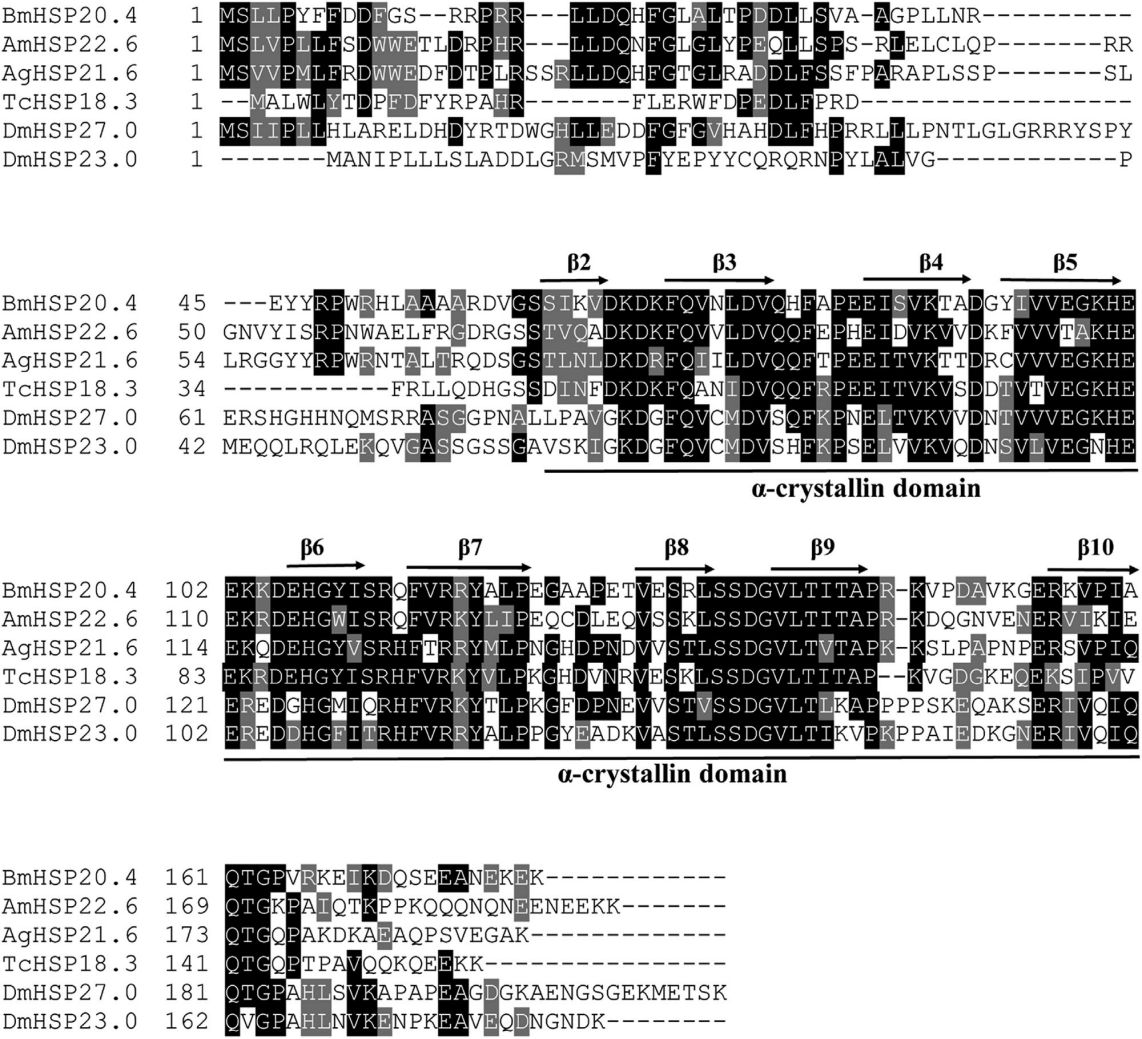
Amino acid sequence alignment of *Tchsp18.3* and five selected *shsps* from *Bombyx mori*, *Apis mellifera*, *Anopheles gambiae* and *Drosophila melanogaster*. Identical residues in the sequences are in black and similar residues are in gray. Secondary structures, as indicated by arrows (β‐sheets) above and line (α‐crystallin domain) below the alignment, were predicted and numbered according to Aevermann and Waters (2008) and Li *et al*. (2009).

Previous annotations for the gene encoding *hsp18.3* in the *T. castaneum* genome (Li *et al*., 2009) was confirmed to be correct by full‐length cDNA cloning and sequencing in this study. *Tchsp18.3* contains a 474 bp open reading frame (ORF) and locates on the LG8 chromosome. The predicted amino acid sequence of *Tchsp18.3* exhibited 40.6%, 41.0%, 42.5%, 32.3% and 30.8% identity to *hsp21.6* (XP_308606) from *Anopheles gambiae*, *hsp22.5* (XP_001119884) from *Apis mellifera*, *hsp20.4* (NP_001037038) from *Bombyx mori*, *hsp23.0* (NP_523999) and *hsp27.0* (NP_524000.1) from *D. melanogaster*, respectively. These *shsps* were the highest sequence identity screened out from other insects; all of them contained the strictly conserved α‐crystalline domain, which consists of approximately 100 amino acid residues and nine common *β*‐strands, with mild to low degrees of conservation in the remaining sequences. Identity of the α‐crystalline domain ranged from 45.4% to 61.5% between *Tchsp18.3* and other *shsps* (Fig. 1).

### The temporal and spatial expression patterns of Tchsp18.3

**Figure 2:**
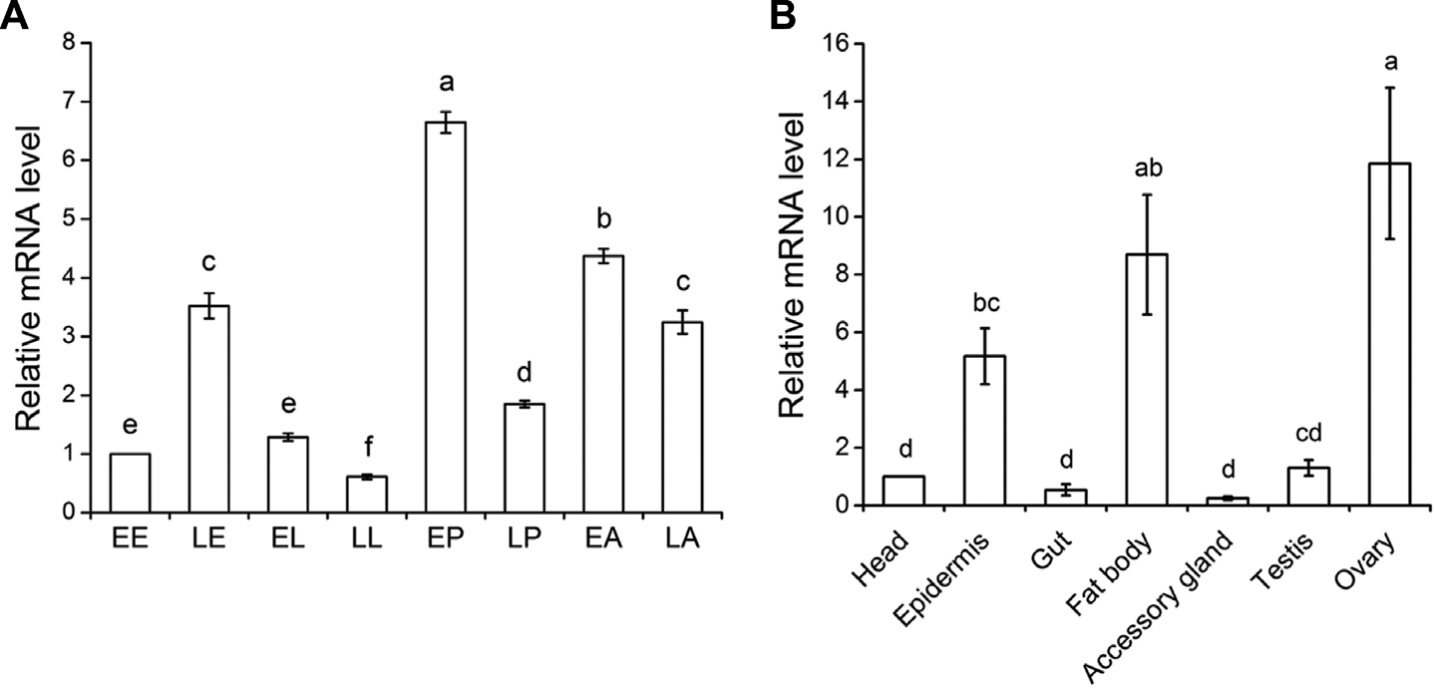
The expression patterns of *Tchsp18.3* in different developmental stages and various late adult tissues. (A) Different developmental stages are EE: early eggs; LE: late eggs; EL: early larvae; LL: late larvae; EP: early pupae; LP: late pupae; EA: early adults; LA: late adults. (B) Various tissues from late adults are head, epidermis, gut, fat body, accessory gland, testis and ovary. *Tribolium* ribosomal protein S3 (*rps3*) transcript with the same complementary DNA (cDNA) template served as an internal control. Different lowercase letters indicate statistically significant differences (*P* < 0.05).

Expression patterns of *Tchsp18.3* were examined by real‐time qPCR. During all stages of development, *Tchsp18.3* was expressed but displayed different expression patterns. It exhibited high expression levels at the early period of each developmental stage except the embryonic stage, while it was expressed the least at late larval stage and the most at the early pupal stage. Data showed that the expression of *Tchsp18.3* at early pupal stage was 10.9 times more than that at the late larval stage (Fig. 2A), while the expression of *Tchsp18.3* in the ovary showed the highest level, which was 49.7 ± 3.7‐fold higher than that in the accessory gland. Moreover, fat body also exhibited abundant expression of *Tchsp18.3* (Fig. 2B).

### Responses to temperature stress

**Figure 3:**
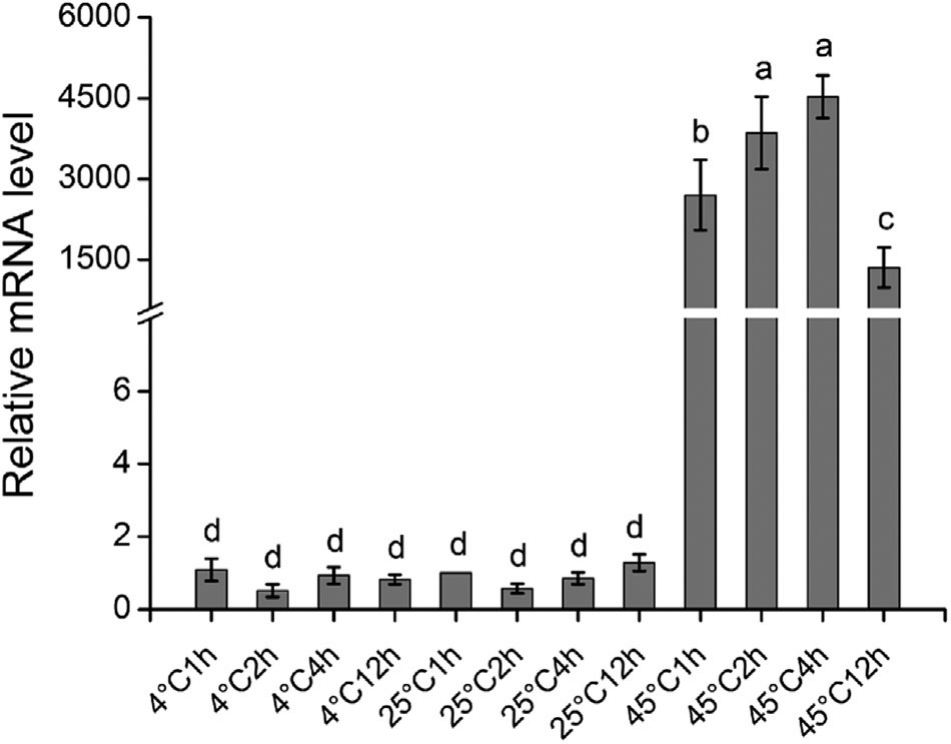
The expression patterns of *Tchsp18.3* under cold (4°C) and heat (45°C) treatment, with an ambient temperature 25°C serving as control. *Tribolium* ribosomal protein S3 (*rps3*) transcript with the same complementary DNA (cDNA) template served as an internal control. Different lowercase letters indicate statistically significant differences (*P*<0.05).

After heat stress, *Tchsp18.3* expression level was significantly increased and it reached a peak after 4 h of treatment at 45°C, which was 4532 ± 396‐fold higher than that without the thermal treatment. Under 45°C for 12 h, the expression level was reduced but still much higher than that of control. Interestingly on the other hand, *Tchsp18.3* did not show any response to cold stress (4 °C) (Fig. 3).

### Responses to starvation stress

**Figure 4:**
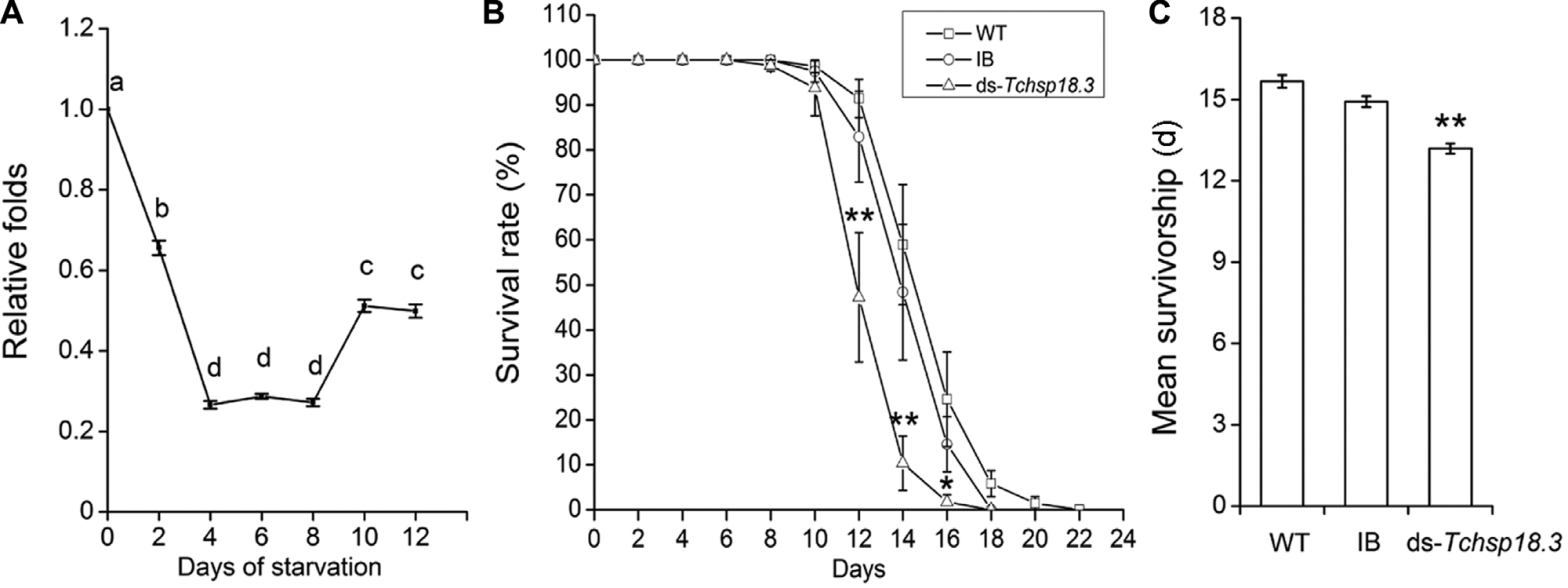
Effect of *Tchsp18.3* on starvation treatment. (A) Time course analysis of *Tchsp18.3* expression under starvation stress. Different lowercase letters indicate statistically significant differences (*P* < 0.05). (B) The differential survival rates of wild type (WT), IB and ds‐*Tchsp18.3* beetles under starvation. (C) Mean survivals (days) of WT, IB and ds‐*Tchsp18.3* beetles under starvation. WT, wild type, beetles received a non‐injection; IB, beetles injected with physiological buffer; ds‐*Tchsp18.3*, beetles injected with double‐stranded RNA in 2‐day‐old pupae. *P < 0.05; **P < 0.001.

After parental RNAi experiment, *Tchsp18.3* gene has been strongly inhibited (Fig. S1A). Under starvation stress, *Tchsp18.3* expression levels were significantly inhibited compared with the WT group until the beetles’ dying, and the relative expression levels were the lowest on the 4th day to the 8th day (Fig. 4A). From the 8th day, adults in the ds‐*Tchsp18.3* group began to die, and the survival rate was significantly lower than the WT group and IB group from 12 days later (Fig. 4B). Finally, adults in the ds‐*Tchsp18.3* group could only survive for 13.2 ± 0.2 days under starvation. It was significantly shorter than the WT group at 15.7 ± 0.2 days and IB group at 14.9 ± 0.2 days, which reduced by 15.8% compared to the WT group (*P* < 0.001) (Fig. 4C). These results showed that the beetles decreased the resistance to starvation when *Tchsp18.3* was knocked down.

### RNAi phenotypes of Tchsp18.3

**Figure 5:**
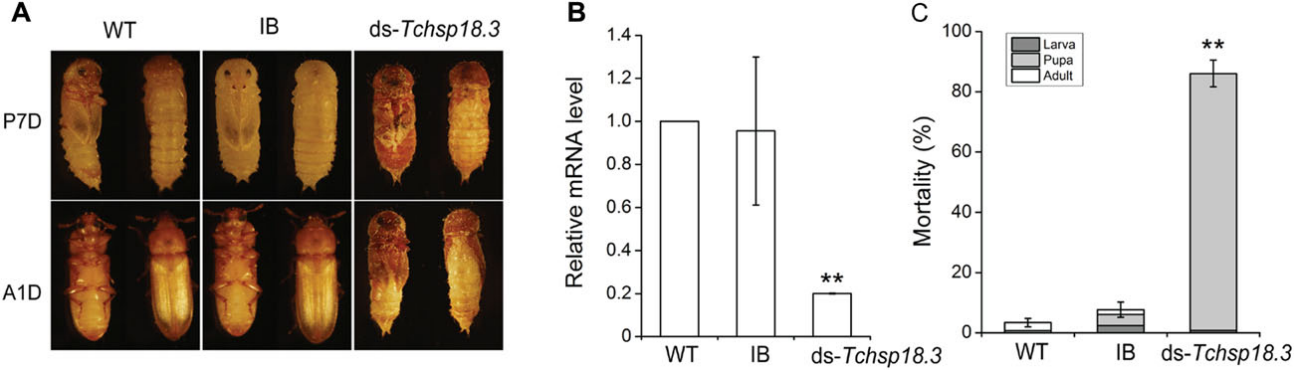
RNA interference (RNAi) phenotype of *Tchsp18.3*. (A) RNAi resulted in lethal phenotypes in pupal/adult stages. P7D, 7‐day‐old pupae; A1D, 1‐day‐old adult. (B) Expression levels of knocking down *Tchsp18.3* using larval RNAi. (C) Mortality of wild type (WT), IB and ds‐*Tchsp18.3* beetles. WT, wild type, beetles received a non‐injection; IB, beetles injected with physiological buffer; ds‐*Tchsp18.3*, beetles injected with double‐stranded RNA in late larval stage. **P < 0.001.

Injection of dsRNA of *Tchsp18.3* during the late larval stage resulted in stagnation or defect in pupal‐adult metamorphosis (Fig. 5A) and suppression of transcript level for *Tchsp18.3* (Fig. S1B; Fig. 5B). Interestingly, larval RNAi of *Tchsp18.3* did not affect the insects’ pupation, but caused approximately 86.1% ± 4.5% of pupae to fail in eclosion (Fig. 5C). These insects with abnormal metamorphosis died at no more than 3 days.

**Figure 6:**
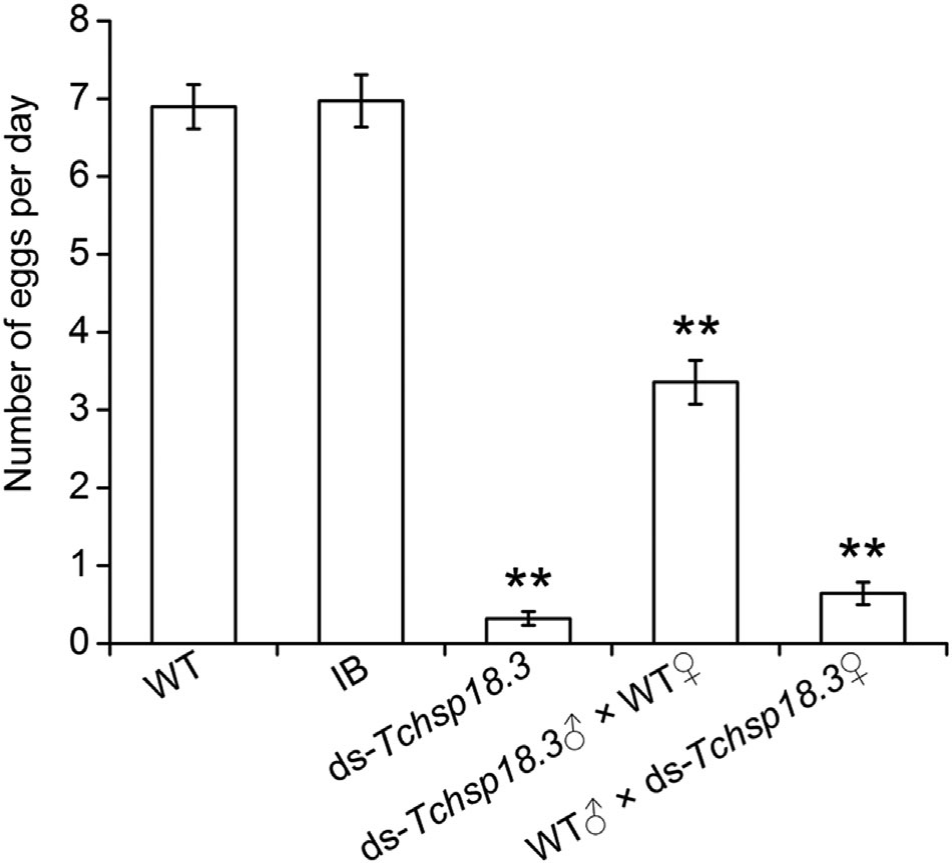
Effect of *Tchsp18.3* on fertility. Wild type (WT), beetles received a non‐injection; IB, beetles injected with physiological buffer; ds‐*Tchsp18.3*, beetles injected with double‐stranded RNA in 2‐day‐old pupae. ds‐*Tchsp18.3*♂ × WT♀, ds‐*Tchsp18.3* male crossed with WT female adult; WT♂ × ds‐*Tchsp18.3*♀, WT male crossed with ds‐*Tchsp18.3* female adult. **P < 0.001.

To investigate the effect of *Tchsp18.3* on reproduction, parental RNAi was carried out. Five groups were set as follow: WT group, IB group, ds‐*Tchsp18.3* group, ds‐*Tchsp18.3*♂ × WT♀ group and WT♂ × ds‐*Tchsp18.3*♀ group. One pair of adults of the WT group and IB group can lay an average of 6.8 and 6.6 eggs per day, respectively. When *Tchsp18.3* was knocked down, one pair of the beetles can hardly spawn. The inhibited oviposition was also discovered when the female and male insects were injected separately, as one pair of beetles in the ds‐*Tchsp18.3*♂ × WT♀ group and WT♂ × ds‐*Tchsp18.3*♀ group can only lay about 3.4 eggs and 0.6 eggs per day, respectively (Fig. 6).

## Discussion

Although sHSPs are probably the most diverse in structure and function among the various superfamilies of stress proteins, they still have relatively conserved domains, the α‐crystallin domain. Usually, the amino‐terminal extension is variable in length and sequence, while in addition to the α‐crystallin domain, the region of the initial 29 amino acids in the amino‐terminal among these six aligned sHSPs showing 73.7% consensus is highly conserved than that of ten sHSPs in *T. castanuem* which only showed 40.7% consensus (Fig. 1). This domain is highly hydrophobic which can be involved in modulating oligomerization, subunit dynamics, and substrate binding (Sun & MacRae, 2005). The relatively higher homology of these sHSPs in the amino‐terminal suggests that *Tchsp18.3* may also conserve in the structure as well as its function among differential organisms.

sHSPs have been shown to play important roles in insect development and always vary according to developmental stage. Here, *Tchsp18.3* showed high expression levels at the early period of each developmental stage except the embryonic stage, and reaching a peak in the early pupal stage (Fig. 2A). The phenomenon of pupal high expression of *shsps* also can be found in other species, for instance, *hsp19.5* in *Plutella xylostella* (Sonoda *et al*., 2006), *hsp19.5*, *hsp20.8* and *hsp21.7* in *Liriomyza sativa* (Huang *et al*., 2009). *Hsp23*, *hsp26* and *hsp27* mRNA of *D. melanogaster* are barely detectable in early third instar larvae but are major components of late third instar and early pupal stages (Mason *et al*., 1984). In the pupal stage, many tissues and organs are degraded and reconstructed during insect metamorphosis. It is likely that metamorphosis itself can serve as a factor to induce the expression of heat shock protein genes (Huang *et al*., 2009). This biological process was further regulated by steroid hormone 20E, which acts through a canonical nuclear receptor complex composed of the ecdysone receptor (EcR) and ultraspiracle (USP) heterodimer (Morrow & Tanguay, 2012). *Cis*‐acting elements involved in the ecdysterone regulation of several *shsp* genes have been mapped, and short sequence elements in the *Dmhsp27* and *Dmhsp23* promoters have been identified as binding sites for EcR (Amin *et al*., 1991). This suggested that expressions of *Dmhsp27* and *Dmhsp23* were regulated in part by the ecdysone hormone which regulated the binding of EcR to ecdysone response elements on the upstream of *shsp* genes. These results indicated that *Tchsp18.3* may be like that of *Dmhsp27* and *Dmhsp23*; its up‐regulated expression during early pupal stage may also involve the hormone regulated metamorphosis. Simultaneously, our RNAi experiment provided strong evidence that *Tchsp18.3* is critical to pupal–adult metamorphosis (Fig. 5A). It showed about 86.1% ± 4.5% failure rate of adult eclosion in the ds‐*Tchsp18.3* group, which indicated that *Tchsp18.3* had a significant effect on metamorphosis and even the survival ability of the beetles.

Expression patterns of insect *shsps* usually show tissue specificity. In *Oxya chinensis*, *hsp19.1*, *hsp20.4*, *hsp20.7* and *hsp21.1* were more highly expressed in the ovary or testis than in the other tissues; meanwhile, the highest expression levels of *hsp19.8* and *hsp23.8* were found in the muscles (Kou *et al*., 2016). In *B. mori*, *hsp19.1* and *hsp22.6* showed relatively high expression levels in integument, head and midgut, while *hsp20.1*, *hsp20.4* and *hsp27.4* were highly expressed in ovary and testis (Li *et al*., 2009). Similar to *hsp20.4* from *B. mori*, *Tchsp18.3* showed the highest expression in ovaries than in other tissues. It was also highly expressed in fat body. These *shsps* expressed in special tissues without stress may play an important role in keeping the normal development of the cell and tissues. For example, *Dmhsp27*, which was highly expressed in ovaries in both unstressed and stressed conditions, played important roles in ovaries either related to the control of cell division/differentiation of germ cells during normal growth, or to the maintenance of ovarian integrity under environmental stresses (Marin and Tanguay, 1996). *Hsp27* in the Mediterranean fruit fly, *Ceratitis capitata*, also was expressed in a stage and cell‐specific manner during oogenesis and spermatogenesis (Economou *et al*., 2017). Interestingly in our study, knockdown of *Tchsp18.3* which had the highest expression in ovaries could cause a significantly declined reproductive capacity (Fig. 6), and since females in the ds‐*Tchsp18.3* group appeared to have significant ovarian atrophy, *Tchsp18.3* acted as a maternal effect gene which was similar to *Tchsp90*. RNAi of both of them led the female to have no offspring, and the male to have low reproduction rates (Zhang *et al*., 2014a). These phenomena implied *Tchsp18.3* may also be involved in the oogenesis and early embryo development as it is likely involved in cell division/differentiation of germ cells during the reproductive process in *T. castaneum*. In addition, higher expression of *Tchsp18.3* in fat body may also be another factor to affect reproduction. Fat body is not only a dynamic tissue involved in multiple metabolic functions and undergoes major functional changes during development and metamorphosis, but also an important site to synthesize and secrete insect yolk proteins, or vitellogenins (Hagedorn & Fallon, 1973). Further, fatty acids stored in the lipid droplets of fat body are mobilized for the provision of lipids to the ovaries, and then maintain the metabolic activity of the organ, because lipids comprise 30%–40% of the dry weight of insect oocytes and they are the main source of energy for the developing embryo (Arrese & Soulages, 2010). Thus, ds‐*Tchsp18.3* affected the normal function of fat body and perhaps could impede the synthesis of vitellogenins or the accumulation of lipid in the ovaries, then declining the reproductive capacity as well.

Moreover, *Tchsp18.3* was dramatically up‐regulated by heat stress. The relative expression of *Tchsp18.3* in 45°C was approximately 4500‐fold higher than that of 25°C, which is much more significant than most *shsps* in other species. Meanwhile, this up‐regulation was more significant than *Tchsp90* in response to heat stress (Zhang *et al*., 2014a). This is in agreement with recent findings. When the cells were is exposed to 40°C heat shock for 1 h, although *Tchsp18.3*, *Tchsp68a* and *Tchsp83* were significantly up‐regulated as a similar expression pattern, *Tchsp18.3* seems to be the most affected (Garcia‐Reina *et al*., 2017). This phenomenon could also be found when beetles were under mild heat shock; *Tchsp18.3* was up‐regulated higher than *Tchsp68* (Altincicek *et al*., 2008). However, *Tchsp18.3* was insensitive to cold treatment (Fig. 3). It is worth mentioning that this is quite different from other structurally similar sHSPs. For example, both *Dmhsp23* and *Dmhsp27* were up‐regulated during recovery from cold stress (Colinet *et al*., 2010), and in *Sarcophaga crassipalpis*, *hsp23* was developmentally up‐regulated during overwintering pupal diapause, the RNAi of which in older pupae even would cause a significant loss of cold tolerance (Rinehart *et al*., 2007). The different reactions to cold of *shsps* may be related to the specific living environments of the insects. Since *T. castaneum* is basically living in a warm cereal storage place and without diapause, then *Tchsp18.3* gene only showed a highly sensitive response to high temperature stress. While Dipteran *D. melanogaster* exhibits diapause at low temperatures and short day lengths (Lumme *et al*., 1974; Saunders *et al*., 1989), it is necessary for *D. melanogaster* to contain certain *shsps* to resist cold stress. These results suggest that the insects as well as their chaperone molecules, *shsp* genes, could evolve according to their own survival environments and requirements.

In addition, when *Tchsp18.3* was knocked down, the beetles decreased the ability to resist starvation, and all adults died within 18 days after starvation treatment. Their average lifespan was significantly shorter than the WT group (Fig. 4C). Meanwhile, *Tchsp18.3* was significantly down‐regulated under starvation conditions, which is consistent with the early finding that a significant reduction in starvation resistance was associated with knockout *hsp27* allele in *D. melanogaster* (Hao *et al*., 2007). In response to starvation, eukaryotic cells re‐absorb nutrition through autophagy, which occurs in the comprehensive reorganization of cellular activities aimed at surviving low nutrient levels (Scott *et al*., 2004). In mammals, phosphorylated dimers of HSP27 can block apoptosis induced by activation of the death receptor *fas* (Charette & Landry, 2000). It further intervened in modulating cell death pathways by interacting with various components of the cell death machinery upstream and downstream of the mitochondrial apoptotic events and can prevent apoptosis in lethal stress situations (Kanagasabai *et al*., 2010; Acunzo *et al*., 2012). Here, knockdown of *Tchsp18.3* was associated with a significant decrease in response to starvation and insect survival rates, suggesting that it may also protect cells from the lethal condition perhaps mainly by its involvement in cell death pathways. Moreover, heat shock proteins could be immunoregulatory agents with potent and widely applicable therapeutic uses (Pockley, 2003). A sophisticated regulation of gene expression in response to different immune stimuli was determined in *T. castaneum*, and *Tchsp18.3* was one of the immune‐induced genes (Altincicek *et al*., 2008). Thus under starvation, down‐regulated expression of *Tchsp18.3* may cause in beetles a decrease in immunity, then dying faster than the control group.

## Conclusion

In conclusion, the temporal and spatial expression patterns, and functions of *Tchsp18.3* have been clarified in the present study. *Tchsp18.3* showed high expression in early pupal stage and was the highest expressed in the ovary and followed the fat body. Knockdown of *Tchsp18.3* in late larvae affected the pupal–adult metamorphosis, which led to 86.1% ± 4.5% of pupae failing in eclosion. Additionally, *Tchsp18.3* silencing caused a significant reduction in reproduction in *T. castaneum*. These results indicated that *Tchsp18.3* keeps certain similarities in expression patterns and several functions with other *shsps*, such as *Dmhsp23* and *Dmhsp27*. However, *Tchsp18.3* also differentiated from them. In addition to response to starvation, it was only strongly stimulated by high temperature stress but not in response to cold stress, which is likely to be an evolutional adaptation to survival environments and habits.

## Disclosure

The authors declare that there is no conflict of interest.

## Supporting information


**Fig. S1** RNA interference efficiency after knockdown experiment in pupal stage (A) and larval stage (B).
