# Supplementary figures and images for: Characterization and functional analysis of hsp18.3 gene in the red flour beetle, Tribolium castaneum
Source: Insect Sci. 2017 Dec 7;26(2):263–73. doi: 10.1111/1744-7917.12543 (PMC7379568; doi:10.1111/1744-7917.12543)

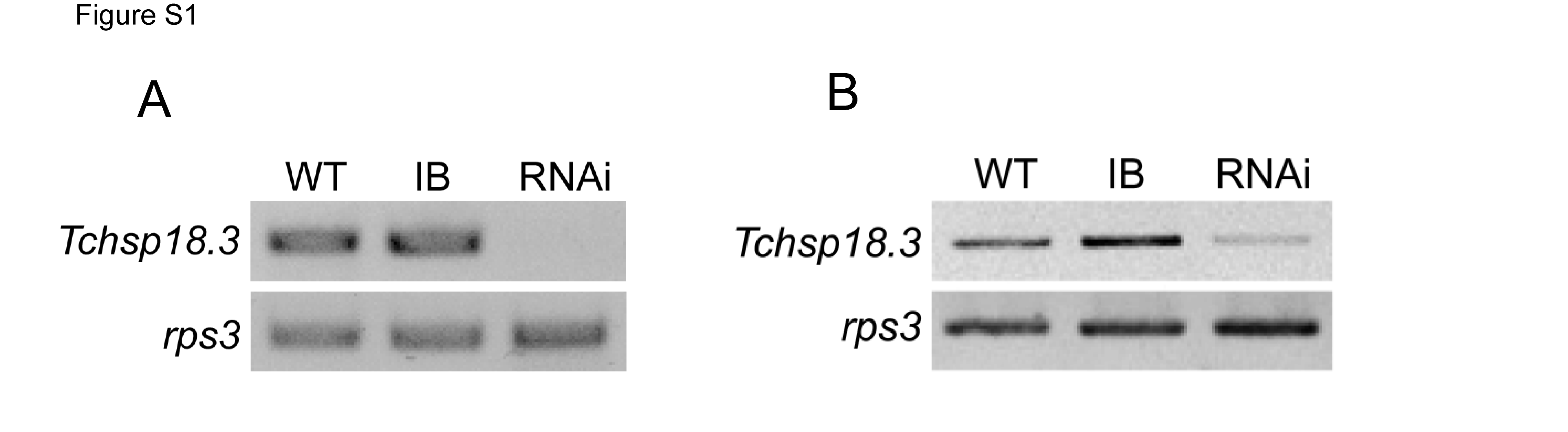

Supplement: Supplementary file 1 — Fig. S1 RNA interference efficiency after knockdown experiment in pupal stage (A) and larval stage (B). [file INS-26-263-s001.tif]
